# Does environmental engineering help rural industry development? Discussion on the impact of Taiwan’s “special act for forward-looking infrastructure” on rural industry development

**DOI:** 10.1007/s11356-020-11059-6

**Published:** 2020-10-03

**Authors:** Chin-Hsien Hsu, Hsiao-Hsien Lin, Shang-Wun Jhang, Tzu-Yun Lin

**Affiliations:** 1 Department of Leisure Industry Management, National Chin-Yi University of Technology, Taichung, 41170 Taiwan; 2 Division of Neurosurgery, Department of Surgery, Changhua Christian Hospital, Changhua, Taiwan; 3 Graduate School of Leisure and Exercise Studies, National Yunlin University of Science and Technology, 123 University Road, Section 3, Douliou, Yunlin 64002 Taiwan

**Keywords:** Public construction, Groundwater pollution, Dust emission

## Abstract

This study investigated the impact of environmental engineering on existing venues, venues and operations management. First, the literature analysis and field survey method are used to summarize the current situation of the venue. Then, 420 questionnaires are collected for statistical verification analysis, combined with the interview method to understand the deepest feelings of the people, and finally discussed with a multi-inspection method. The researcher believes that although environmental construction can improve infrastructure and human quality of life, which, after all, are experiment after completion. If, before construction, a good communication channel; obtaining consensus from the people and businesses; acquiring precise information; spraying water to reduce dust; increasing the height of the peripheral fence; planning a substitutive plan; avoiding crowds; reducing mistakes in the process; avoiding influencing the water and electricity supply and soil, water, and environment sanitation; and planning for a substitutive route and parking space with subsidy for damages are not possible, a negative image will be produced, willingness to spend will be reduced in the people due to the blocking of the view by the building (75%), the billboard will lose its functionality (63.2%), and inconvenience will be caused by the line of movement (75%) and parking space (55.9%), which are not helpful for development.

## Introduction

National public construction revitalizes the economy and improves the development status (Lu 2019; Yu 2005), increases job opportunities, and promotes universal health (Lin 2019). On the basis of the “One Town One Product” policy in Taiwan, various public construction projects such as stadiums have been proposed. This provides safe and suitable recreational environments for the public, thereby enhancing overall public construction development. After public construction, facility management requires professional cooperation, but after the Taiwan government builds public construction, the commission is managed by local government units. However, the management personnel of local government agencies depend on familiarity with the national examination books. After passing the examination, they can be appointed as public officials. However, such a manpower source is only familiar with the contents of the book, seriously lacks professional technology and practical experience, and is not competent for technical administrative management tasks. As a result, due to the lack of management talents in public construction, the problem could not be resolved. In the long run, countless venues and venues were idle (Yao 2016).

To resolve the problem of idle buildings, government agencies have planned to outsource business processes to trustees to create a win–win situation for the government and businesses (Hsu 2002; Mihelcic et al. 2006; Reible 2017). However, most public construction projects lacked reasonable planning policies and have failed to consider three aspects, namely systems, implementation, and professionalism (Wang 2018), because the regulating authority lacks professionalism and human resources and has poor operating ability (Ye 1991). Therefore, the government intends to attract professional teams and businesses to use and revitalize idle venues and fields by outsourcing. Businesses that manage these properties should have enthusiasm and corporate social responsibility to earn consumers’ recognition (Fang 1998; Hsu 2003). Moreover, these two characteristics help businesses obtain lucrative opportunities, operate smoothly, and create a win–win situation for the government, venues and fields, and the general public, thereby achieving sustainable development (Lin 2019). Therefore, efficient goal attainment must be enhanced for construction projects.

National development policy includes social, economic, and environmental aspects (Ministry of Internal Affairs of the Republic of China 2020). In the national public construction, environmental engineering is also one form of public decision, all having the effect of enhancing development in towns and villages (Chung et al. 2013; CHEN 2019). There is a diverse way for intervention of environmental engineering, in which there is revitalization of existing space or facilities in old buildings by reorganizing or rebuilding after redesigning (Fu 2017). The construction site, due to reformation by environmental engineering, combining, integrating, and planning with existing resources, from which the space or merchandise is created as a result result(Mackenzie and David 2010), can produce new value that can be utilized by the local people to promote economic development (LAI 2019). It is apparent that with good public policy planning, the environmental engineering can help in the development of towns and villages.

Environmental engineering is the direction and measure of public policy (LAI 2019) that is mainly applying science and engineering to improve the environment, due to which the people’s expectations are satisfied (Chung et al. 2013), and a living space fit for humans is provided (Wang 2018). However, under the policy, the impact after environment engineering construction is not only in the change of space or construction but also changes in the natural ecology and environment (Shen 2017). Therefore, the implementation of environmental engineering must meet the need of the people in order to achieve the real expectation of the society and the people. Related decisions are shown in Table 1.

**Table 1:** Public works ecology verification mechanism

Classification	Explanation
Subject	Alleviate the negative impact on ecology by public works with principles of ecology preservation, civic participation, and open information to actively create a quality environment
Aims	Post-disaster emergency handling, emergency repair, emergency rescue, post-disaster reconstruction, planning for ecology verification necessary for new building construction besides acquiring the construction of green buildings and related maintenance and management
Features	The ecology verification is divided, based on the construction life cycle, to approval of the construction plan, planning, design, construction, and maintenance stages. Each construction plan, at the design, construction, and maintenance stages, complies with the ecology protection strategy for conducting verification. Set an ecology verification mechanism that complies with the characteristics of the construction of the agency and combine verifications on the different stages.

Urban development should shift focus from development to management (Water Environment Research Center 2006). Construction planning should be step by step and draw from international experience, while adjusting the local pace and valuing public participation and realization in order to meet the need of the country and society (Water Environment Research Center 2006; LAI 2019) and acquire real public construction value as well as establish the goal of sustainable ecological city construction. However, the information shows that the state of recent public constructions or environmental engineering is geared toward the development of tourism (Lin et al. 2017) or environmental endangerment (Dong 2019). Those with environmental engineering as the theme for understanding the impact on the existing environment and facility by the construction are rare. Also lacking are the collecting of public experience and researches on the impact on the existing environment and facility by environmental engineering. Therefore, the researcher believes that an objective answer for whether the result of environmental engineering is beneficial or has a negative impact can be derived from people’s point of view looking at the impact on the existing facility and environment by environmental engineering.

The government proposed the “one characteristic per town/village, one swimming pool per city” project in the hope of providing a safe water leisure and sports environment to the public while developing a local leisure industry to increase business opportunity. As shown in Table 2, in order to avoid the failure of venue activation, the government formulates a private participation in the public construction bill, hoping to use private investment and operation to activate idle venues. However, besides setting a good policy, local government also requires the spirit of policy insistence to maintain the consistency of the policy to lower damage to the environment, provide reasonable investment return for the businesses, enhance the management capability of contract dispute and crisis, fortify the receiving system, and establish a good cooperation relationship between the government and the private sector in order to promote the effectiveness of the private sector in participating in public construction bills (Chen et al. 2017). Therefore, having a complete planning and policy communication is the key to the effectiveness of public construction execution and sustainable development.

**Table 2:** Taiwan “Act for Promotion of Private Participation in Infrastructure Projects”—case status review

Case	Defect
Taichung Intercontinental Baseball Stadium	The plan lacks prior evaluation and creates controversy.
Taoyuan National Sports Center	The official venue management unit lacked communication with the public and did not conduct academic evaluations and investigations, resulting in public misunderstandings.

**Fig. 1:**
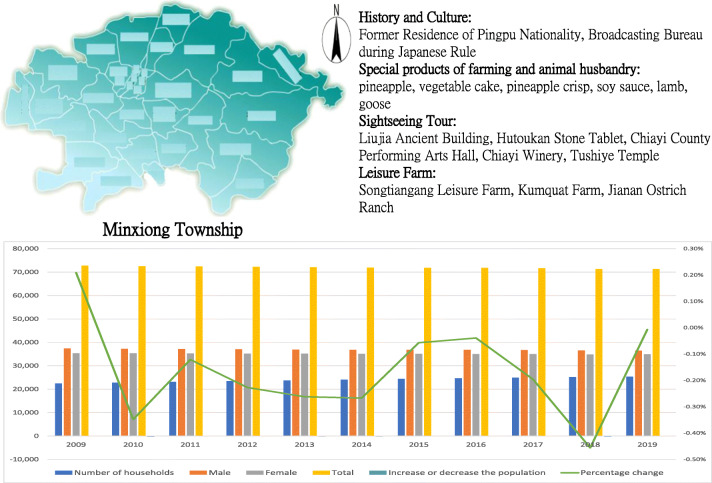
Analysis of the development status of Minxiong Township

Minxiong Township is located in the mid-northern part of Chiayi County in Taiwan, with a population of over 50 thousand people (2020, Ministry of Interior). It is the third largest township in the country with terrains of hills and plains, full of agricultural development potential. It is rich in agricultural and husbandry products. Moreover, it was under Japanese control. Minxiong Township of Chiayi County is an old Taiwanese village with rich historical culture and agricultural industry resources, as shown in Fig. 1. Because of its aging population, the village’s economic development has been hindered, and its available human resources for environmental maintenance have decreased.

In order to promote the national leisure sports and improve people’s quality of life, Minxiong Township is building a swimming pool and looks forward to introducing the leisure industry and revitalizing the local economy. Minxiong Township has completed and actuated the sports park swimming pool which has been in existence for 25 years. Although the supervisory unit is dedicated in coordinating and operations management, attempting to develop the leisure industry, however, due to lack of manpower and professionalism, the result has not been good. In 2001, the open commissioning bid was won by Oceania Swimming Pool Equipment Ltd. for 9 years. However, due to various reasons, they forfeited the operations right after the end of the contract, resulting in an idle pool. From people’s imploring and policy supervision, the business management unit has allotted a budget in 2012 for operation. Yet, due to lack of professional human resources and funding, operations ended after 4 months from deficit, as shown in Table 3.

**Table 3:** Overview of Minxiong Township sports park swimming pool operating units and management effectiveness

Year	Operators	Business month	Operating days	Profit and loss
Before 2000	Oceania Swimming Pool Equipment Co., Ltd.	A whole year	9 years	–
2012	Minxiong Township Office	Only from July to September	83 days	$1 million loss
2016		Only from April to October	6 months	Monthly profit and loss—96,000, annual hydropower cost budget—2.19 million
2017	You Falcon Sports Co., Ltd.	Only from April to October	About 9 months	Monthly profit and loss—76,000, current profit and loss + 1.43 million
2018		A whole year (renovated in 3.4 months)	About 10 months	Monthly profit and loss—42,000, the current profit and loss + 1.77 million

**Fig. 2:**
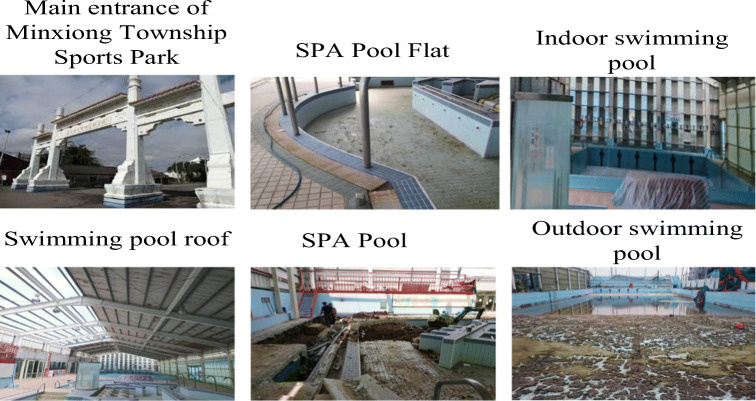
Minxiong Township Sports Park swimming pool—before 2016

There have been several operating units, including that from the public department, since the opening of the site. However, bad management, lack of funding, manpower, and professionalism still lead to the result of having the site idle, as shown in Fig. 2.

**Fig. 3:**
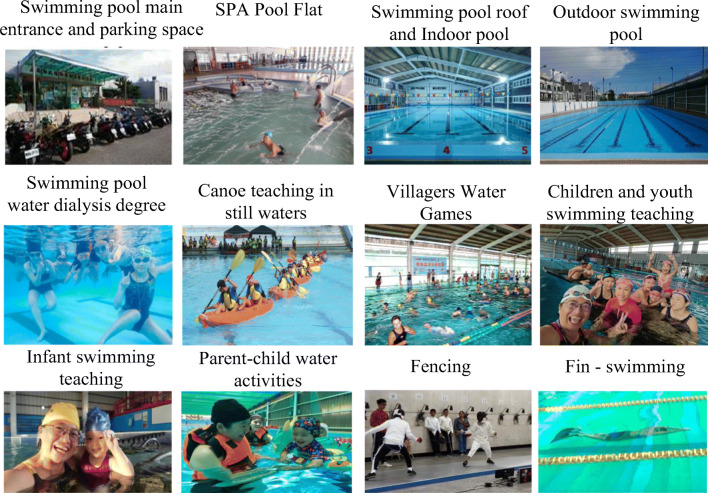
Status of the swimming pool operation management and development—from 2017 to the present

With the correction by the Directorate-General of Budget, Accounting, and Statistics of Executive Yuan, the supervisory unit has found a professional team with which a 5-year commissioning contract was signed in 2017 to take over the task of managing the Minxiong Township Sports Park swimming pool. The company used 2 years with high standard of management brought in diverse water course programs, exquisite basic training, dedication in offering swimming lessons, promoting water safety experience for the people, holding fun water contests that showed results of gradual annual growth. According to statistics, there was a total of 239,765 swimmers and participants in classes (You Jhun Sprot Business Co 2018). As compared to 12,683 in 2017, the sports site has been successfully revitalized (Chiayi County Audit Office 2017), as shown in Fig. 3.

The revitalization of venues not only solves the lack of local government governance but also promotes the effectiveness of the municipal government’s decision-making and moves to the next stage. The local government formulated the Minxiong Forest Plan of Minxiong Township of Chiayi County, the goal of which is to improve the cityscape and residents’ quality of life and start construction to revitalize the old downtown area (Chiayi County Government 2019). The initial proposal failed because numerous local public facilities have been poorly operated and have been idle for several years. A renovation project subsidized with $216 million was approved after said facilities were successfully revitalized after improvement (Lin and Lee 2019; Ministry of the Interior 2018a, b).

**Fig. 4:**
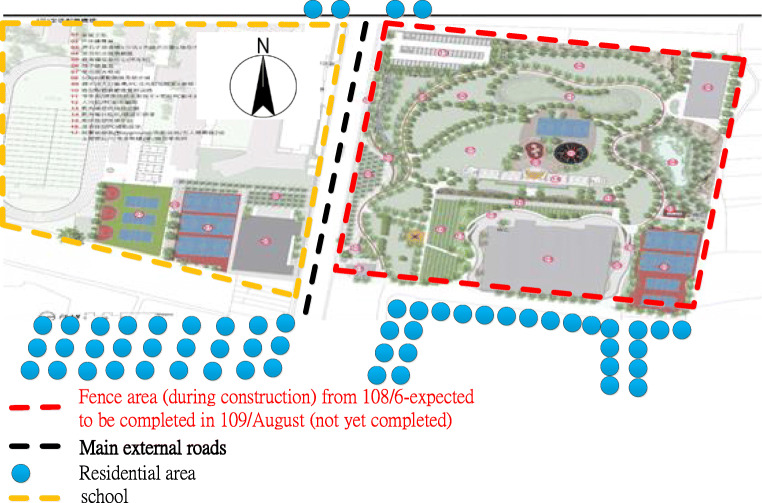
Description of construction scope of special act for forward-looking infrastructure

Over 3 years from 2018 to 2020, the subsidized project has reconstructed all existing parks except for those that were successfully revitalized by outsourcing management. The aim of this project is to create inclusive and compound landscapes for playgrounds and sports fields (Pan Yu Arcuitects and Associates 2018). To meet industrial safety requirements, construction fences were installed, as shown in Fig. 4.

Even though environmental engineering is an engineering science, in response to the need or fault of local development, it is through engineering science and technology from the angle of social science that efforts are made to improve the people, businesses, and objects, and eliminate the environment dilemma, resulting in a better community environment and public construction, providing better water, air, and soil for the people. However, the final goal of policy planning is to satisfy the needs of humans and improve the economy, society, and current state of the environment, and environmental engineering policy is using environmental engineering technology from the angle of science to improve the current development of villages and to meet the expectations of the people. Thus, environmental engineering is not just the science of environmental management and engineering technology; its impact is related to social science. It is a science of a combination of different fields of science and also a form of public policy.

Usually, the result of the policy can only be assessed after the completion of a plan (Ap and Crompton 1998; Jurowski et al. 1997; Gursoy et al. 2002). Yet, the best assessors are the users and local residents(Lankford and Howard 1994; Paulo and Pinto 2017). The result will be most objective and deep from the reactions of both to determine the effectiveness of the policy (Anderson 1990; Lin et al. 2018). This can elucidate the effect of public construction and implementation conditions on current venues, facilities, and business operators, and will facilitate decision-making. As a result, the researcher believes that reviewing environmental engineering policy from the viewpoint of the people will provide a deeper understanding of the actual result and problem of the policy in order to arrive at a more perfect policy planning.

Commissioned operations of idle sites should be expected to create a win–win situation for the government and businesses (Xu 2002). However, the reality is that an overall lack of consideration, bad marketing, lack of a good system, execution, and professionalism (Yang 2018a, b) lead to lack of communication during execution and lack of forward thinking in terms of professionalism and integrated and systemic thinking. The engineering plan is still in the form of having an order coming from the top (Yang 2018a, b), which resulted in the inevitable fate of idleness due to lack of integration and the inability of the business or supervisory unit to promote the site (Xu 2002).

However, continual construction errors have caused problems such as water and power outages that affect facility maintenance and management, which reduced visitors to swimming pools by 200% (You Jhun Sprot Business 2019). This indicates that major construction plans and management systems in Taiwan lack care consideration. Specifically, Taiwan’s professionalism in constructing and implementing projects lacks communication and progressive, integrative, and comprehensive ideas. Construction plans remain in a top-down administration and management process (Lin and Lee 2019). Without comprehensive planning and consideration, conflicts between project promotion and facility management can occur, and the project would fail to meet its goals (Hsu 2002; Yang 2018a, b). Therefore, the study believes that exploring the construction process and the impact of individual cases can help to understand the effectiveness of environmental engineering decision-making.

## Materials and methods

**Fig. 5:**
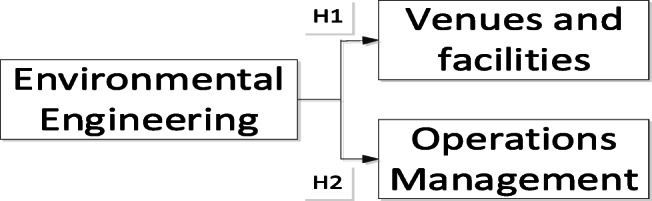
Research framework

We investigated current venues, facilities, and business operation conditions in the Minxiong Forest Plan of Minxiong Township of Chiayi County to reveal the effect of environmental engineering on these venues and their business operations, in Fig. 5.

### Research scope and tools

We conducted field observation, collected data in person, and edited our interview and questionnaire topics on the basis of relevant studies (Chiayi County Government 2019; Dixon et al. 2013; Fang 1998; Hsu 2002, 2003; Lin 2019; Lin and Lee 2019; Lu 2019; Mihelcic et al. 2006; Ministry of the Interior 2018a, b; National Audit Office of Taiwan, Chiayi Office 2017; Pan Yu Arcuitects and Associates 2018; Reible 2017; Wang 2018; Yang 2018a, b; Ye 1991; You Jhun Sprot Business Co 2019; Yu 2005). A pretest was conducted using 200 questionnaires in June 2019 (Gorsuch 1983; Wu and Tu 2009), and items with correlation coefficients greater than 0.4 were adopted (Wu and Tu 2009).

In reference to the aforementioned literature, 23 questions for venues and facilities (Hsu 2003) (9), marketing and service quality (Lin and Lee 2019) (9), operations management(Fang 1998) (5) are edited. After completion, content validity is examined, after which 50 questionnaires are collected and analyzed with SPSS for Windows 22.0 statistics software. When KMO > 0.08, and Bartlett’s *p* value < 0.01; it means the scale is fit for ensuing factor analysis (Kaiser 1974); furthermore, with an *α* coefficient over 0.80, it means the questionnaire has good reliability (Devellis 1991).

From the analysis, there are 9 questions for venues and facilities (Hsu TK 2003), the result of analysis for which is KMO of 0.932 and Bartlett *χ*2 of 19,977.650, df of 58, significance of *p* < 0.001, suitable for factor analysis. The explained variability of the scale is 42% and that of combined explained variation is 42%, all of which are kept after factor analysis. The *α* coefficient is 0.976–0.978.

There are 9 questions on marketing and service quality (Lin and Lee 2019). The result of the analysis is KMO of 0.931, Bartlett χ2 of 19,977.650, df of 58, significance of *p* < 0.001, suitable for factor analysis. The explained variability of the scale is 39%, combined explained variation is 43.12%, all of which are kept after factor analysis. α coefficient is 0.977–0.978.

There are 5 questions on operations management (Fang 1998). The result of the analysis is KMO of 0.922, Bartlett *χ*2 of 1825.10, df of 463, significance of *p* < 0.001, suitable for factor analysis. The explained variability of the scale is 39%, combined explained variation is 43.12%, all of which are kept after factor analysis. The *α* coefficient is 0.977–0.978. Based on the above results of the analysis, it is derived that the compiled questionnaires on the issues of venues and facility, marketing and service quality, and operations management have good validity and are fit for ensuing utilization and investigation, as shown in Table 4.

**Table 4:** Results of reliability test and factor analysis

Issue		M	SD	Cronbach’s *α*
Venues and facilities	Entrance signs and parking planning	3.79	0.886	0.978
	Traffic flow planning in venues and fields	4.06	0.762	0.977
	Poolside space planning	4.24	0.703	0.976
	Resting and storage space	4.17	0.756	0.977
	Swimming pool water quality and temperature management	4.26	0.686	0.977
	Design of poolside area, sewer covers, and stairs	4.05	0.885	0.977
	Shading and ventilation planning	4.02	0.868	0.976
	Toilet equipment	3.89	0.897	0.977
	Willingness to revisit the swimming pool	4.00	0.804	0.976
Marketing and service quality	Current signboard placement	3.86	0.821	0.977
	Print advertisements in venues and facilities	3.91	0.759	0.977
	Current Internet marketing management	3.92	0.829	0.977
	Current lifeguards’ professionalism and responsibility	4.21	0.755	0.977
	Current reception staff’s professionalism and responsibility	4.27	0.775	0.977
	Current swimming coaches’ professionalism and responsibility	4.35	0.774	0.978
	Influence on teaching environment and quality	4.27	0.775	0.977
	Influence on consumption environment and quality	4.26	0.751	0.978
	Willingness to revisit the swimming pool	4.29	0.674	0.977
Operation management	Planning of current teaching sites	4.24	0.766	0.977
	Planning of available teaching content	4.36	0.757	0.976
	Current teaching environment and facilities	4.41	0.803	0.976
	Current teaching staff and professional technology	4.38	0.907	0.976
	Willingness to participate in swimming activities again	4.30	0.877	0.976

The research adopts triangulation for exploration that can enhance the structure of the literature (Janesick 2000; Anselm et al. 1998; Gursoy et al. 2002). Combining literature analysis, field investigation, statistical verification, and peer verification mechanism is established to interpret information and explore with diverse viewpoints (Janesick 2000). Firstly, using SPSS for Windows 22.0 is used to statistically analyze and verify sample data while designing an interview outline. Semi-structured open interviews with 10 local residents, businesses, engineering personnel, and scholars are conducted on their views on the impact on local development generated by environmental engineering (as in Table 2). Finally, the diverse information is compiled (Anselm et al. 1998) for cross checking and verification (Janesick 2000), using, in order, concluding, organizing, and analyzing to derive the final correct and reasonable information to construct the paper (Gursoy et al. 2002), as shown in Table 5.

**Table 5:** Interviewee information

Respondents code	Identity	Gender	Years of swimming	Job title
A	Scholar	Male	20	Lecturer
B	Scholar	Female	42	Lecturer
C	Residents	Male	40	Elder
D	Residents	Female	15	Commissioner
E	Residents	Female	39	–
F	Pool operator	Female	35	Coach
G	Pool operator	Male	40	Lifeguard
H	Pool operator	Female	42	Supervisor
I	Government engineer	Male	5	Engineer
J	Government engineer	Female	3	Engineer
Interview outline				
1. Whether environmental engineering has an impact on the management of the existing environment and facilities, please explain your reasons and views. 2. Whether environmental engineering has an impact on the existing service quality and management, please explain your reasons and views. 3. Whether environmental engineering has an impact on the operation and management of existing industries, please explain your reasons and views.				

### Description of research restrictions

The study focuses on the construction process of the special act for forward-looking infrastructure in Minxiong Township, and explores whether public decision-making and environmental engineering can contribute to the development of rural industries. However, due to the limitations of the project period, scope, and safety of the construction site, only the construction site is the center, the sampling range is spread outward, and the neighboring enterprises, consumers and residents are taken as the sampling objects for analysis and discussion.

## Results and discussion

A total of 420 questionnaires were distributed at the venue, and most participants were women (75% women and 25% men). This suggested that most visitors to the swimming pool are families because in Chinese society, fathers focus on working, and mothers are responsible for children and the family. Consequently, most visitors to the swimming pool are women.

### Analysis of the Effect of Environmental Engineering on the Situation of Venues and Fields

Urban development should shift focus from development to management (Water Environment Research Center 2006). Construction planning should be step by step and draw from international experience, while adjusting the local pace and valuing public participation and realization in order to meet the need of the country and society (Water Environment Research Center 2006; LAI 2019) and acquire the real public construction value, and establish the goal of sustainable ecological city construction.

**Fig. 6:**
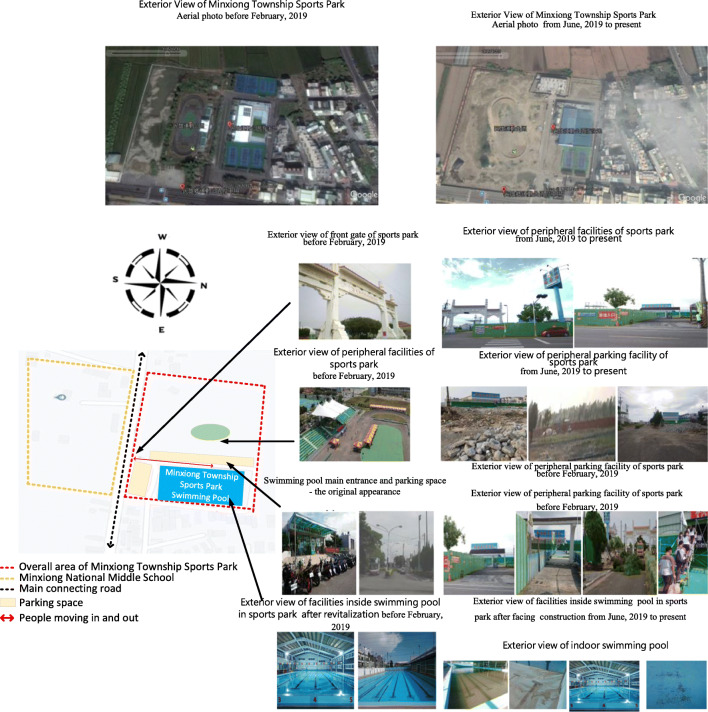
Analysis of differences between Minxiong Sports Park and swimming pool before and after construction

The survey found the following: There was originally a good space in the sports park swimming pool facility and its surrounding environment; however, after construction, the exterior and space layout, traffic routes, and parking space changed. The main hardware structure underwent adjustment, and dust fallout was severe. Air quality changed, and with it, the appearance of water quality is also affected, as in Fig. 6.

**Fig. 7:**
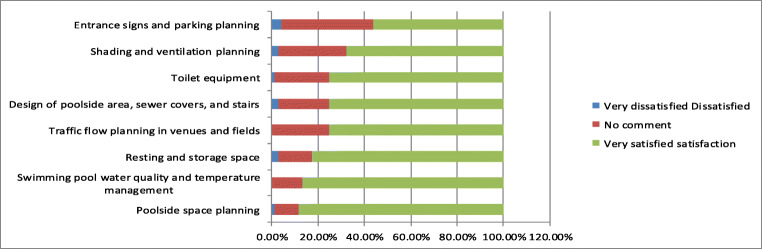
Analysis of the people’s cognition on the current situation of the maintenance of sports facilities

As shown in Fig. 7, the general public expressed that businesses are thoughtful and maintain excellent quality in water, resting places, and traffic flow planning. Moreover, 75% of respondents were willing to reuse the businesses’ services. However, they expressed concern in using their services because of the following drawbacks: frequent transportation of large construction vehicles, walls blocking the pool’s surroundings and closed access roads, uncertain access road safety, difficulty locating the swimming pool, outdated indoor insulation boards, and inadequate planning of outdoor shading. Even with high-quality physical environments inside and outside venues and excellent maintenance of other environmental aspects such as water quality, other factors can discourage people from using the swimming pool.

**Fig. 8:**
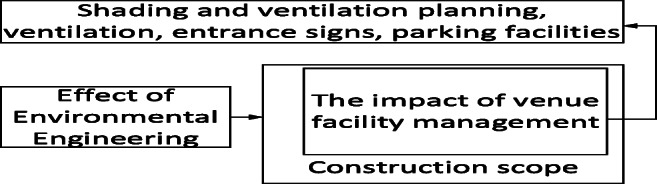
Impact of environmental engineering on site management

Specifically, drawbacks including flawed traffic flow and dust emissions caused by environmental engineering construction, transportation of large construction, obstruction of traffic flows to the swimming pool, old and damaged equipment, and unfavorable ventilation and shading design undermined respondents’ approval of maintenance and operation management at venues and fields. This reduced their willingness to reuse their services, as shown in Fig. 8.

The process of environmental engineering requires the assistance of big heavy machinery and transport vehicles, during which there would be a major impact on the existing site and surrounding environment. The researcher thus believes that the constructing party needs to have due diligence on communication and obtain the agreement of the local residents and businesses while providing compensation. Spray water to reduce dust fallout and plan for alternate road and signs to reduce damage.

### Analysis of the impact of environmental engineering on current marketing and service quality

Environmental engineering refers to the use of scientific methods and decision-making to promote construction, improve the local life and public leisure environment, and provide high-quality water, soil, air, and other living environments; the people get a safe and friendly living space and the local economy can develop. People living and living in peace is the goal of public decision-making (Wang 2010; Fu 2017; Shen 2017).

**Fig. 9:**
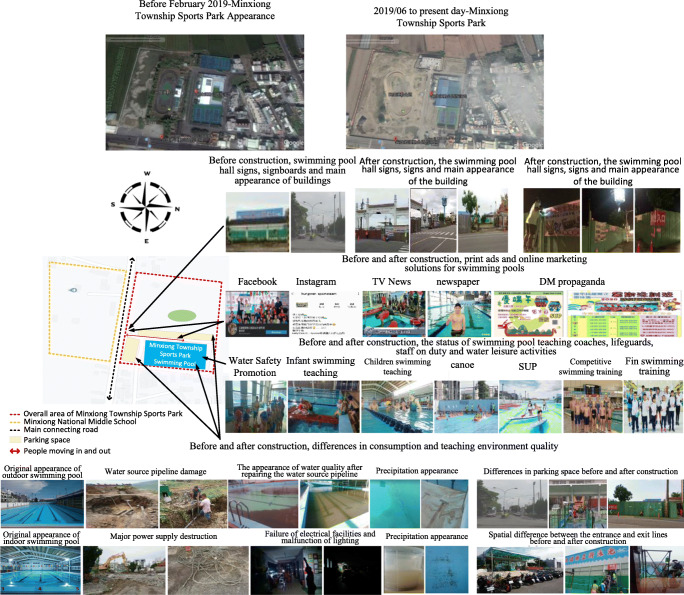
Difference analysis of the impact of environmental engineering on sports facilities operating facilities and marketing and service quality

The survey found that after the swimming pool has been revitalized, the operator has aggressively improved the equipment, maintained high water quality and sanitation, promoted swimming lessons to the public, promoted water safety and self-rescue activities, and further introduced diverse on-the-water and under-the-water activity equipment and classes, all of which have gained good reviews by the consumers, creating 239.7 visitors (You Jhun Sprot Business Co 2018). However, since the construction began in June 2019, big construction machinery and trucks enter and exit frequently. The traffic route and parking space have greatly changed. The surrounding view is blocked by the construction site, affecting the exterior appearance of the swimming pool site and the water quality while construction mistakes that appeared continually destroying water and electric equipment are making a direct and indirect impact on the existing main structure and operations management, as in Fig. 9.

**Fig. 10:**
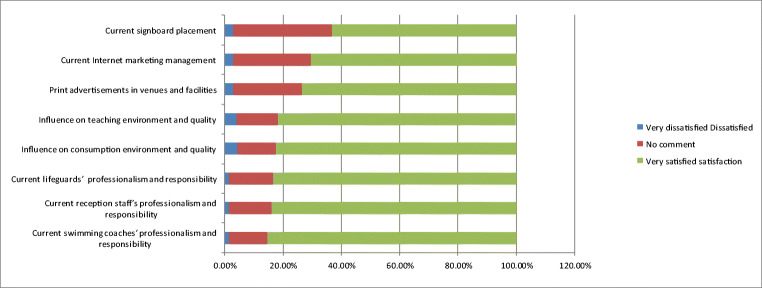
Cognitive analysis of the impact of environmental engineering on current marketing and service quality

As shown in Fig. 10, the majority of respondents (88.2%) were willing to reuse their services indicating that businesses have high standards for their operation strategies, maintaining employees’ dedication to their work, and maintaining an excellent corporate image and corporate social responsibility. However, the public could not gain knowledge of the current operation management of swimming pools because of fences blocking the road, reduced traffic flow and spaces, and difficulty identifying the current entrance to the swimming pool. Construction misinterpretation caused damage to cables and water pipelines, and they could not provide water or electricity. Broken underground pipelines can be polluted and lead to considerable amounts of sewage, sediment, and underground wastewater flowing into the pool. This increases the burden of water quality management and accelerates filtration system impairment, resulting in unfavorable water quality and appearance of the swimming pool.

**Fig. 11:**
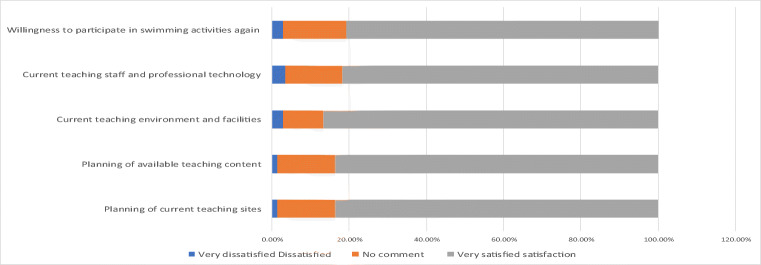
Cognitive analysis of the effect of environmental engineering and operation management

The general public approved of teaching sites, teaching content, teaching staff, and professional technology, and 80.9% of visitors were willing to revisit (Fig. 11).

**Fig. 12:**
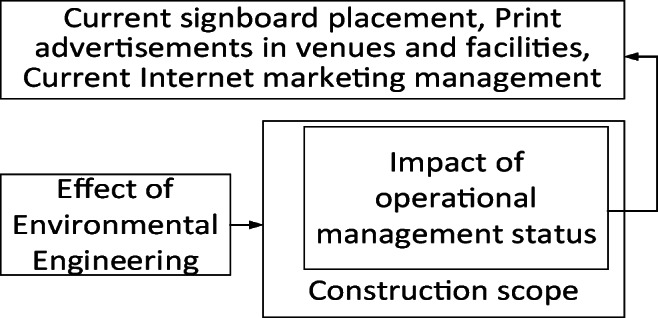
Impact of environmental engineering on operational management

Safety maintenance measures required in environmental engineering, such as demolition and transportation, and unfamiliarity with on-site construction environments have impaired facilities and caused difficulties for operation managers, as shown in Fig. 12.

Construction operations may result in damages from mistakes rendering change in the peripheral environment or buildings, and feeling threatened in the local resident. The researcher thus believes that before construction, the construction party needs to conduct a thorough field investigation and communicate with or visit local residents and businesses in order to obtain the most accurate and detailed on-site information. Moreover, they need to prepare for response measures and establish complaint channels, machinery equipment, or subsidy to prevent and reduce loss when construction mistakes occur.

### Operation management and annual difference in the number of visitors

Urban development should pay attention to the feelings of the people (LAI 2019), maintain the consistency of policies to reduce damage to the environment, provide reasonable investment returns for enterprises, enhance the management capacity of contract disputes and crises, and shift the focus from development to management (Water Environment Research Center 2006) to improve the effectiveness of the private sector participation in public construction bills (Chen et al. 2017) and implement the goal of sustainable development.

The survey found businesses grew by an average of 120% between 2017 and 2018, but growth was reduced by up to 200% because of the aforementioned reasons (Ministry of the Interior 2018a, b; You Jhun Sprot Business Co 2018) (Table 6).

**Table 6:** 2017, 2018, and 2019 statistical differences in the number of tourists from July to September (person-times)

Date	2017	2018	2017–2018 growth rate	2019	2018–2019 attrition rate
July	926	1632	176.24%	585	– 279.10%
August	802	1013	126.31%	440	– 230.20%
September	378	754	199.47%	258	– 265.10%

The general public believed that businesses strive to train athletes, can plan competent teaching content and have professional and technical skills, and devote themselves to environmental protection. However, the swimming pool’s surroundings had considerable amounts of dust and water, and a power outage occurred once. Additionally, a construction company mistakenly damaged an underground water pipeline, causing sewage inflow that undermined the quality of the water and environmental appearance. Moreover, sludge flowing into the pond increased the burden on the filtration system and undermined its function. Overall, this caused unfavorable energy waste, hygiene, and appearance at the swimming pool.

**Fig. 13:**
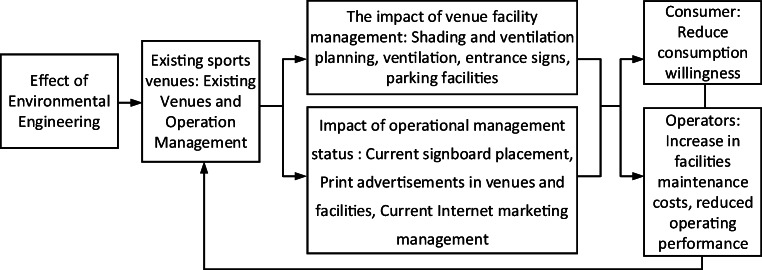
The effect of environmental engineering on existing venues and operation management

Problems such as dust emissions and pollution can discourage people from revisiting the pool and reduce the number of visitors even if businesses are effective in operation, marketing, and management and maintain professional teaching and service quality, as shown in Fig. 13.

The generation of dust fallout and air pollution will lower people’s quality of life, endanger their health, and be a nuisance to the residents or businesses in the surrounding area. The researcher thus thinks that during construction, the constructing party must implement stringent water spraying, increase the height of the surrounding fence, avoid peak hours to reduce dust fallout, and block the expansion of the dust in order to acquire the approval of the local residents and businesses and the result of the construction enhanced.

## Conclusion

Environmental engineering can contribute to economic, social, and environmental development. However, if decision-makers cannot reach a consensus with businesses and resolve problems such as reducing dust emissions, correcting fence planning, adjusting transportation routes, improving site survey accuracy, and reducing engineering errors, numerous negative outcomes will result. Even when businesses are effective in operation, marketing, and management and maintain professional teaching and service quality, the condition of the venues’ tangible and intangible facilities would be influenced. This discouraged people from reusing services, reduced the number of visitors, and undermined effective operation management.

Based on the above survey results, the research recommendations are as follows:

1. Obtain approval from residents or neighboring companies before construction and give compensation.
2. Conduct field surveys before construction to obtain accurate and detailed on-site information.
3. Plan alternative roads and signs to reduce damage.
4. Prepare contingency measures and set up complaint pipelines, machinery equipment, or subsidies to prevent losses when engineering errors are caused.
5. Implement watering measures, increase the height of surrounding fences, and avoid peaks of crowds to reduce falling dust and prevent the spread of dust.
6. Based on the results of this study, expand the investigation of other topics to complete the research information.

## Data Availability

All data were collected with the consent of the interviewees, and the analysis data was obtained from the analysis after the research team collected samples. All data and photos were obtained from the research subjects, and the photos were released in the content of this research after their consent.
